# Impact of heat stress during close-up dry period on performance, fertility and immunometabolic blood indices of dairy cows: prospective cohort study

**DOI:** 10.1038/s41598-024-72294-2

**Published:** 2024-09-11

**Authors:** Barbara Stefanska, Ewa Pruszynska-Oszmalek, Veerle Fievez, Cezary Purwin, Włodzimierz Nowak

**Affiliations:** 1 Department of Grassland and Natural Landscape Sciences, Poznań University of Life Sciences, 60-632 Poznan, Poland; 2 Department of Animal Physiology, Biochemistry, and Biostructure, Poznań University of Life Science, 60-637 Poznan, Poland; 3 Laboratory for Animal Nutrition and Animal Product Quality (Lanupro), Department of Animal Sciences and Aquatic Ecology, Ghent University, 9000 Gent, Belgium; 4 Department of Animal Nutrition, Feed Science, and Cattle Breeding, University of Warmia and Mazury, 10-719 Olsztyn, Poland; 5 Department of Animal Nutrition, Poznań University of Life Sciences, 60-637 Poznan, Poland

**Keywords:** Global warming, Climate change, Temperate climate, Metabolic adaptation, Energy balance, Immune response, Immunology, Physiology

## Abstract

This study aimed to investigate whether heat stress, as defined by the temperature-humidity index (THI) during the close-up dry period, had any impact on the productive performance, fertility, and immunometabolic blood indices of dairy cows in the subsequent lactation. Lactation performance was associated with increasing THI values on − 21, − 14, and − 7 d before calving resulting in decreased milk yield by about 2.30, 2.60, and 2.90 kg, respectively. The THI on the − 7 d before the calving was negatively associated with fertility parameters such as delayed first estrus postpartum, an elongated calving interval by approximately 32 d, a higher number of services per conception by 1.00, and an elongated artificial insemination service period, days open, and inter-calving period by about 20, 52, and 52 d, respectively. The study found that the immunometabolic blood indices were associated with increasing THI values during the close-up dry period. The study showed that exposing dairy cows to close-up dry period heat stress had negative consequences on performance, fertility, and immunometabolic blood indices in the subsequent lactation. Therefore, it is recommended that herd management and barn microclimate changes be implemented earlier, starting from the late dry period, to mitigate the negative impact of heat stress.

## Introduction

Global warming presents a significant threat, not only to human health worldwide but also to livestock production. Reports from international and government research centers have consistently shown a trend toward the systematic warming of the Earth’s climate^1^. If climate change continues unabated, it is projected that temperatures could increase by as much as 4 °C by the year 2100^2^. This poses a substantial challenge for cattle breeders, as dairy cows are highly sensitive to changing environmental conditions, including rising air temperatures (AT) and relative humidity (RH). This sensitivity arises from their elevated metabolic heat production associated with rumen fermentation and hepatic nutrient metabolism^3^. The combination of increased metabolic heat production and climate changes leads to the occurrence of heat stress (HS), a physiological condition in which the total heat load, comprising both environmental and internal heat production, surpasses the capacity for heat dissipation. This condition results in decreased thermal comfort for the cows^4^. When the upper critical AT and RH are exceeded, the adaptive mechanisms of the cows fail to effectively dissipate the excess heat generated^1^. To more accurately assess thermal HS, bioclimatic indices such as the temperature-humidity index (THI), which integrates AT and RH have been developed^5^. Various authors have proposed different THI threshold values, typically ranging from 68 to 74 units^6^, depending on the climatic zone. A THI of 68 is frequently used as an indicator of HS in both lactating^7^ and dry cows^8^ in temperate climate zones^9,10^. This threshold was derived from a series of eight studies where the milk yield of 100 high-producing Holstein cows decreased by 2.2 kg/day for each 24-h period at a daily THI of 68. While the biological mechanisms underlying HS during the late dry period remain unclear^11,12^, it is believed that these metabolic alterations account for approximately 50% of the observed milk production losses^13^, as well as an increased number of days open and services per conception in the subsequent lactation^14^. Therefore, accurate prediction of HS and identifying periods of its greatest negative impact are crucial for maintaining the welfare, performance, and fertility of dairy cows. This becomes increasingly important due to the predicted rise in the frequency of high-temperature days associated with climate change and the high sensitivity of dairy cows to HS^15^. Currently, there is limited scientific data available on the carry-over effects of HS, as evaluated by THI, also from Eastern Europe in temperate climates during the critical transition period, where the dairy industry is an important part of livestock production^16^.

The transition from the nonlactating to the lactating state can be a challenging period for dairy cows and is associated with significant physiological changes due to negative energy balance (NEB) and heightened immune dysfunction^17^. These changes have adverse effects on the health, milk production, and reproductive performance of cows. Around calving, inflammation is commonly observed in nearly all cows, often stemming from tissue remodeling and bacterial infections^18^. Inflammatory dysfunction impacts the metabolic status of dairy cows by increasing lipolysis and leading to higher circulating levels of non-esterified fatty acids^19^. HS also induces disruptions in nutrient metabolism in the liver similar to those observed during NEB in the transition period. Researchers are currently investigating biomarkers that can help elucidate the biological mechanisms connecting HS and NEB with the immunity of dairy cows during the transitional period after calving. These mechanisms may affect performance and fertility during lactation^12^. While THI is a valuable tool for assessing HS, it does not account for individual variability. Immune indices such as interleukin 1 (IL-1), interleukin 6 (IL-6), and tumor necrosis factor-α (TNF-α) might be explored as potential additional markers to confirm the HS status of animals^20^. Therefore, it will be interesting, to analyze the physiological changes during the close-up dry period under condition HS, because they might have an impact on the immunometabolic status of dairy cows in the subsequent lactation. Understanding the effects of HS during the close-up dry period could be instrumental in making informed decisions regarding farm management during this critical phase. Implementing interventions such as providing shade to block direct solar radiation, using water spraying to enhance conductive heat loss, and improving ventilation to facilitate evaporative cooling could prove beneficial in mitigating the consequences of HS and subsequently enhancing milk yield, fertility performance, and immunometabolic status in dairy cows during the subsequent lactation.

In this present study, we hypothesized that HS during the close-up dry period would be associated with a decline in productive performance, fertility, and alterations in the immunometabolic blood indices of dairy cows in the subsequent lactation. The objective of this study was to assess whether HS, as determined by the THI during the close-up dry period, had any impact on the productive performance, fertility, and immunometabolic blood indices of dairy cows in the subsequent lactation.

## Results

### Lactation performance and body condition scores

In the current study, as THI increased on the − 21, − 14, and − 7 days before the calving, milk yield decreased by approximately 2.30, 2.60, and 2.90 kg, respectively (Tables 1, 2, 3). Conversely, a decrease in THI on the − 21 d before calving resulted in higher milk fat and protein contents (*P* ≤ 0.01). However, increasing THI values on the − 14 and − 7 d before calving day were associated with the highest percentage of milk fat (*P* ≤ 0.01). Increasing THI values on all analyzed days during the close-up dry period were associated (*P* ≤ 0.01) with an increase in milk SCC. No significant association was observed between THI values during the close-up dry period and MU concentration.

**Table 1:** Impact of the temperature-humidity index (THI) − 21 d before calving on dairy cows’ lactation performance and body condition score.

Item	THI –21 d			SEM	*P-value*			*P-value*/Estimate (± 95% CI)	
	< 68	68–72	> 72		Farm (*H*_i_)	Dam parity (*L*_j_)	THI − 21 d^1^ (*B*_k_)	AFC^2^ (β_1_afc_l_)	DIM^3^ (β_2_dl_m_)
Milk yield (kg)	30.1^A^	29.8^AB^	27.8^B^	0.28	< 0.0001	0.1297	0.0027	0.4954/− 0.0069	0.1020/− 0.0237
Milk fat (%)	4.10^A^	3.95^AB^	3.88^B^	0.01	< 0.0001	0.2228	0.0028	0.8858/− 0.0001	0.0700/− 0.0004
Milk protein (%)	3.53^A^	3.46^AB^	3.39^B^	0.01	0.0002	0.3121	0.0021	0.2329/− 0.0005	0.0002/− 0.0024
SCC (10^3^ cells/mL)	198^B^	256^AB^	332^A^	0.06	0.0075	0.1252	0.0082	0.4498/0.0021	0.8802/− 0.0004
MU (mg/mL)	197	215	226	4.24	< 0.0001	0.8895	0.8895	0.9317/− 0.0068	0.0152/0.3229
^4^Milk fatty acids (FA), g/100 g fat									
C14:0	11.8	11.6	11.3	0.05	< 0.0001	0.2347	0.2377	0.9510/− 0.0001	< .0001/− 0.0200
C16:0	29.5	29.5	30.1	0.10	0.0002	0.8723	0.0773	0.7931/− 0.0009	< .0001/− 0.0283
C18:0	9.53	9.80	9.85	0.06	0.0285	0.4736	0.2739	0.8508/− 0.0003	< .0001/− 0.0285
C18:1	18.8	18.8	19.5	0.13	0.0106	0.3724	0.5716	0.6635/− 0.0371	< .0001/− 0.0021
SFA	66.1	65.8	65.2	0.14	0.0209	0.5491	0.0649	0.7121/− 0.0019	< .0001/0.0312
UFA	27.8	27.6	28.6	0.14	0.0251	0.2826	0.1729	0.7319/− 0.0018	< .0001/− 0.0411
MUFA	22.7	22.7	23.0	0.13	0.0310	0.8277	0.6175	0.9730/− 0.0001	< .0001/− 0.0335
PUFA	2.96	2.90	2.82	0.02	0.2353	0.4579	0.0589	0.5513/− 0.0005	0.3227/− 0.0012
TRANS_FA	1.74	1.51	1.54	0.05	< 0.0001	0.2594	0.9019	0.5276/0.0010	0.0030/− 0.0067
SCFA	10.6	10.2	9.93	0.05	0.8483	0.4342	0.3140	0.8233/− 0.0003	0.2018/− 0.0029
MCFA	43.8	43.7	42.8	0.19	0.0103	0.2451	0.1105	0.8104/− 0.0016	< .0001/− 0.0478
LCFA	31.4	31.6	32.5	0.19	< 0.0001	0.4628	0.3185	0.9836/− 0.0532	< .0001/− 0.0011
MCFA/SCFA	4.13	4.28	4.31	0.12	0.0158	0.4537	0.2549	0.9510/− 0.0001	< .0001/− 0.0210
SFA/UFA	2.38	2.38	2.28	0.05	0.0172	0.4763	0.3729	0.7931/− 0.0009	< .0001/− 0.0823
DI18	66.4	65.7	66.4	0.02	0.0016	0.4397	0.5275	0.8508/− 0.0003	< .0001/− 0.0257
BCS on the calving day	3.30	3.34	3.42	0.08	0.3583	0.6423	0.4589	0.6635/− 0.0371	0.2527/− 0.0012
BCS 21 d after calving	3.42	3.37	3.43	0.05	0.1158	0.2346	0.4819	0.7121/− 0.0019	0.3421/− 0.0067

^1^THI − 21 d = temperature-humidity index calculated on the − 21 d before the calving and divided into three categories: THI < 68 as a thermal comfort zone (*n* = 31), 68–72 as mild heat stress (*n* = 35), and THI > 72 as heat stress for dairy cows (*n* = 34).

^2^AFC = age first calving.

^3^DIM = days in milk.

^4^Milk fatty acids (g/100 g fat)—SFA = saturated FA; UFA = unsaturated FA; MUFA = monounsaturated FA; PUFA = polyunsaturated FA; TRANS_FA = trans-unsaturated FA; SCFA = short-chain FA; MCFA = medium-chain FA; LCFA = long-chain FA; MCFA/SCFA = medium-chain FA to short-chain FA ratio; SFA/UFA = saturated FA to unsaturated FA ratio; DI18 = the proportion of C18:1 to the sum of C18:0 and C18:1 multiplied by 100.

^A,B^Means of the explanatory variable within the category of THI on the − 21 d before calving within the row with different letters differed significantly (*P* ≤ 0.01).

**Table 2:** Impact of the temperature-humidity index (THI) − 14 d before calving on dairy cows’ lactation performance and body condition score.

Item	THI –14 d			SEM	*P-value*			*P-value/Estimate (± 95% CI)*	
	< 68	68–72	> 72		Farm (*H*_i_)	Dam parity (*L*_j_)	THI − 14 d^1^ (*B*_k_)	AFC^2^ (β_1_afc_l_)	DIM^3^ (β_2_dl_m_)
Milk yield (kg)	30.5^A^	29.2^AB^	27.9^B^	0.28	< .0001	0.1297	0.0065	0.4790/− 0.0068	0.1049/− 0.0232
Milk fat (%)	3.70^B^	3.78^AB^	4.10^A^	0.01	< .0001	0.2228	0.0088	0.8284/− 0.0002	0.6841/− 0.0006
Milk protein (%)	3.47	3.48	3.48	0.01	0.0002	0.3121	0.8539	0.3883/− 0.0003	0.0001/− 0.0025
SCC (10^3^ cells/mL)	243^B^	312^A^	389^A^	0.06	0.0148	0.1252	0.0028	0.9770/− 0.0022	0.2996/− 0.0001
MU (mg/mL)	190	213	222	4.24	< .0001	0.8895	0.9613	0.8298/− 0.0166	0.0058/− 0.3194
^4^Milk fatty acids (FA), g/100 g fat									
C14:0	11.9^a^	11.6^ab^	11.4^b^	0.05	< .0001	0.2347	0.0223	0.5947/− 0.0008	< .0001/− 0.0204
C16:0	29.1	29.6	29.9	0.10	0.0008	0.8723	0.5159	0.4302/− 0.0028	< .0001/− 0.0294
C18:0	9.31	9.79	9.82	0.06	0.0058	0.4736	0.4911	0.5079/− 0.0288	< .0001/− 0.0012
C18:1	18.5	18.8	19.7	0.13	0.0172	0.3724	0.3772	0.5178/− 0.0382	< .0001/− 0.0031
SFA	65.9^A^	65.7^A^	65.1^B^	0.14	0.0016	0.5491	0.0064	0.4776/− 0.0323	< .0001/− 0.0036
UFA	27.7^B^	27.7^B^	28.5^A^	0.14	0.0083	0.2826	0.0040	0.4542/− 0.0038	< .0001/− 0.0427
MUFA	23.3	22.6	22.4	0.13	0.0158	0.8277	0.1993	0.9261/− 0.0345	< .0001/− 0.004
PUFA	2.90	2.97	2.83	0.02	0.1057	0.4579	0.9073	0.4545/− 0.0006	0.3770/− 0.0011
TRANS_FA	1.67	1.74	1.41	0.05	< .0001	0.2594	0.7602	0.6108/− 0.0007	0.0024/− 0.0069
SCFA	10.3^A^	10.2^AB^	9.96^B^	0.05	0.5526	0.4342	0.0028	0.7574/− 0.0004	0.1610/− 0.0032
MCFA	43.9^A^	43.8^A^	42.4^B^	0.19	0.0003	0.2451	0.0025	0.9406/− 0.0004	< .0001/− 0.0491
LCFA	31.6^B^	31.8^B^	32.5^A^	0.19	< .0001	0.2628	0.0012	0.7013/− 0.0548	< .0001/− 0.0026
MCFA/SCFA	4.26	4.29	4.26	0.12	0.0558	0.5537	0.4393	0.9510/− 0.0001	0.4270/− 0.0210
SFA/UFA	2.38	2.37	2.28	0.05	0.0432	0.3763	0.7273	0.2345/− 0.0001	0.5270/− 0.0823
DI18	66.5	65.8	66.7	0.02	0.0326	0.4397	0.5202	0.4308/− 0.0013	0.3431/− 0.0257
BCS on the calving day	3.22^A^	3.14^AB^	2.98^B^	0.08	0.4584	0.3423	0.0016	0.5435/− 0.0172	0.2527/− 0.0012
BCS 21 d after calving	3.38^A^	3.27^AB^	3.05^B^	0.05	0.5283	0.4346	0.0024	0.8221/− 0.0219	0.3421/− 0.0067

^1^THI − 14 d = temperature− humidity index calculated on the − 14 d before the calving and divided into three categories: THI < 68 as a thermal comfort zone (*n* = 31), 68–72 as mild heat stress (*n* = 31), and THI > 72 as heat stress for dairy cows (*n* = 38).

^2^AFC = age first calving.

^3^DIM = days in milk.

^4^Milk fatty acids (g/100 g fat)—SFA = saturated FA; UFA = unsaturated FA; MUFA = monounsaturated FA; PUFA = polyunsaturated FA; TRANS_FA = trans-unsaturated FA; SCFA = short-chain FA; MCFA = medium-chain FA; LCFA = long-chain FA; MCFA/SCFA = medium-chain FA to short-chain FA ratio; SFA/UFA = saturated FA to unsaturated FA ratio; DI18 = the proportion of C18:1 to the sum of C18:0 and C18:1 multiplied by 100.

^a,b^Means of the explanatory variable within the category of THI on the − 14 d before calving within the row with different letters differed significantly (*P* ≤ 0.05).

^A,B^Means of the explanatory variable within the category of THI on the − 14 d before calving within the row with different letters differed significantly (*P* ≤ 0.01).

**Table 3:** Impact of the temperature-humidity index (THI) − 7 d before calving on dairy cows’ lactation performance and body condition score.

Item	THI –7 d			SEM	*P*-value			*P-*value/Estimate (± 95% CI)	
	< 68	68–72	> 72		Farm (*H*_i_)	Dam parity (*L*_j_)	THI − 7 d^1^ (*B*_k_)	AFC^2^ (β_1_afc_l_)	DIM^3^ (β_2_dl_m_)
Milk yield (kg)	30.3^A^	29.6^AB^	27.4^B^	0.28	< 0.0001	0.1297	0.0027	0.9547/− 0.0005	0.1245/− 0.0219
Milk fat (%)	3.57^B^	3.85^AB^	4.29^A^	0.01	< 0.0001	0.2228	0.0079	0.9634/− 0.0001	0.6403/− 0.0007
Milk protein (%)	3.46	3.49	3.49	0.01	0.0012	0.3121	0.4446	0.2757/− 0.005	0.0002/− 0.0025
SCC (10^3^ cells/mL)	234^B^	306^AB^	354^A^	0.06	0.0342	0.1252	0.0063	0.0383/− 0.0045	0.9968/− 0.0001
MU (mg/mL)	203	206	229	4.24	< 0.0001	0.8895	0.9372	0.0065/− 0.3153	0.8105/− 0.019
^4^Milk fatty acids (FA), g/100 g fat									
C14:0	11.9^a^	11.6^ab^	11.4^b^	0.05	< 0.0001	0.4722	0.0210	< .0001/0.0203	0.9586/0.0001
C16:0	29.1	29.7	29.9	0.10	0.0003	0.7238	0.2712	0.8306/0.0008	< .0001/0.0289
C18:0	9.51	9.57	9.91	0.06	0.0040	0.7364	0.3096	0.8520/− 0.0003	< .0001/− 0.0295
C18:1	18.4	19.0	19.4	0.13	0.0019	0.7324	0.1217	0.9658/− 0.0002	< .0001/− 0.0372
SFA	66.6^A^	65.4^B^	65.1^B^	0.14	0.0027	0.4951	0.0036	0.8939/− 0.0007	< .0001/0.0322
UFA	27.1^B^	27.7^B^	28.6^A^	0.14	0.0011	0.2643	0.0038	0.9130/− 0.0005	< .0001/− 0.418
MUFA	22.9	22.9	22.2	0.13	0.0030	0.8721	0.0515	0.4999/0.0033	< .0001/− 0.0337
PUFA	2.95	2.89	2.82	0.02	0.0014	0.5792	0.2234	0.0439/0.0018	0.2351/− 0.0015
TRANS_FA	1.76	1.50	1.47	0.05	< 0.0001	0.2649	0.8460	0.4948/− 0.0068	0.0029/0.0011
SCFA	10.2^A^	10.4^AB^	9.91^B^	0.05	0.8918	0.4238	0.0025	0.8058/0.0004	0.1808/0.0031
MCFA	44.0^A^	43.9^A^	42.1^B^	0.19	< 0.0001	0.4521	0.0015	0.4532/− 0.0050	< .0001/0.0488
LCFA	31.6^B^	31.8^B^	32.7^A^	0.19	< .0001	0.2585	0.0092	0.9087/− 0.0008	< .0001/− 0.0544
MCFA/SCFA	4.31	4.22	4.25	0.12	0.1558	0.3743	0.4393	0.2310/− 0.0032	0.2740/− 0.0210
SFA/UFA	2.46	2.36	2.28	0.05	0.2432	0.6338	0.7273	0.4548/− 0.0054	0.5610/− 0.0823
DI18	65.9	66.5	66.2	0.02	0.2645	0.9278	0.5202	0.4088/− 0.0045	0.5621/− 0.0257
BCS on the calving day	3.24^A^	2.89^AB^	2.76^B^	0.08	0.0584	0.6328	0.0022	0.4355/− 0.0145	0.9857/− 0.0012
BCS 21 d after calving	3.14^A^	2.82^AB^	2.72^B^	0.05	0.0523	0.4634	0.0048	0.8221/− 0.0324	0.6531/− 0.0067

^1^THI − 7 d = temperature-humidity index calculated on the − 7 d before the calving and divided into three categories: THI < 68 as a thermal comfort zone (*n* = 31), 68–72 as mild heat stress (*n* = 31), and THI > 72 as heat stress for dairy cows (*n* = 38).

^2^AFC = age first calving.

^3^DIM = days in milk.

^4^Milk fatty acids (g/100 g fat)—SFA = saturated FA; UFA = unsaturated FA; MUFA = monounsaturated FA; PUFA = polyunsaturated FA; TRANS_FA = trans-unsaturated FA; SCFA = short-chain FA; MCFA = medium-chain FA; LCFA = long-chain FA; MCFA/SCFA = medium-chain FA to short-chain FA ratio; SFA/UFA = saturated FA to unsaturated FA ratio; DI18 = the proportion of C18:1 to the sum of C18:0 and C18:1 multiplied by 100.

^a,b^Means of the explanatory variable within the category of THI on the − 7 d before calving within the row with different letters differed significantly (*P* ≤ 0.05).

^A,B^Means of the explanatory variable within the category of THI on the − 7 d before calving within the row with different letters differed significantly (*P* ≤ 0.01).

Specifically, dairy cows exposed to HS (THI > 72) on the − 14, and − 7 d before the calving exhibited a decrease in the content of C14:0 (*P* ≤ 0.05) as well as SFA, SCFA, and MCFA (*P* ≤ 0.01). Conversely, an increasing THI value at the same analyzed points during the close-up dry period was associated with an increase in the contents of UFA and LCFA (*P* ≤ 0.01). However, no significant association was observed between THI values on the − 21 d before the calving and milk fatty acids profile. Also, no significant association was observed between THI values on the − 14, and − 7 d before the calving and individual milk fatty acids such as C16:0, C18:0, and C18:1, their groups such as MUFA, PUFA, trans-FA, and ratios such as MCFA/SCFA, SFA/UFA, and DI18.

The THI value on the − 14 and − 7 d before calving was associated (*P* ≤ 0.01) with a linear decline in body condition score (BCS) on the calving day and 21 d after calving.

### Fertility and immunometabolic blood indices

The HS during the close-up dry period appeared to be associated with both fertility and immunometabolic blood indices in dairy cows (Tables 4, 5, 6). An increase in THI value on the − 7 d before calving was linked to a delayed manifestation of the first estrus postpartum and an extended calving interval (*P* ≤ 0.05), with each parameter being elongated by approximately 32 days. Additionally, an increase in THI value on the − 7 d before calving was associated (*P* ≤ 0.05) with a decline in fertility indices, including an increased number of services per conception by approximately 1.00, and an elongated AI service period, days open, and inter-calving period by around 20, 52, and 52 d, respectively. However, no significant association was observed between THI values on the − 21 and − 14 d before the calving during the close-up dry period and reproductive performance.

**Table 4:** Impact of the temperature-humidity index (THI) − 21 before calving on dairy cows’ fertility indices and immunometabolic blood indices.

Item	THI –21 d			SEM	*P-*value			*P-*value/Estimate (± 95% CI)	
	< 68	68–72	> 72		Farm (*H*_i_)	Dam parity (*L*_j_)	THI − 21 d^1^ (*B*_k_)	AFC^2^ (β_1_afc_l_)	BSD^3^ (β_2_ds_m_)
Fertility									
First estrus pp, d	51.9	66.4	78.0	1.22	0.4816	0.4395	0.6246	0.4464/0.0034	–
Calving interval, d	72.9	87.4	99.0	3.76	0.3677	0.5132	0.4438	0.3525/0.1561	–
AI period service, d	32.7	40.2	41.5	2.31	0.2251	0.3820	0.5247	0.3164/0.3504	–
Services per conception	1.56	1.91	1.98	0.15	0.1451	0.0800	0.7754	0.3364/0.0224	–
Days open, d	106	128	141	5.37	0.6265	0.9626	0.3860	0.2597/0.2065	–
Inter-calving period, d	381	403	416	6.98	0.4999	0.7626	0.5907	0.1385/0.6757	–
Immunometabolic blood indices									
Insulin, ng/mL	5.47	5.52	5.71	0.09	0.2447	0.0469	0.9725	0.7361/− 0.0010	0.6242/− 0.0091
IGF-I, ng/mL	70.9	70.0	65.9	2.27	0.0598	0.8760	0.7350	0.0316/− 0.1490	0.5137/− 0.2564
NEFA, µmol/l	53.0^b^	53.6^b^	65.9^a^	1.60	0.5131	0.7207	0.0184	0.9807/0.0010	0.4977/0.1799
BHB, mmol/L	5.70	6.54	7.10	0.69	0.5416	0.6395	0.3146	0.7004/− 0.0107	0.7821/0.0459
ApN, μg/mL	5.42	5.34	5.23	0.16	0.7504	0.8258	0.5247	0.0286/− 0.0137	0.9266/− 0.0032
LEP, μg/mL	1.66	1.71	1.84	0.06	0.3596	0.4091	0.6234	0.0227/− 0.0053	0.4413/− 0.0101
FSH, ng/mL	7.15	6.89	6.77	0.14	0.0901	0.6181	0.6002	0.4695/− 0.0025	0.8950/− 0.0028
LH, ng/mL	6.80	6.51	6.41	0.14	0.0834	0.2977	0.6049	0.8845/− 0.0006	0.9349/− 0.0022
TNF-α, ng/L	358^b^	462^b^	523^a^	15.6	0.0772	0.8014	0.0204	0.3500/− 0.5091	0.2731/− 3.5856
IL-1, ng/L	13.6	14.0	15.8	0.59	0.4046	0.8926	0.4981	0.0320/− 0.0368	0.6717/− 0.0411
IL-6, ng/L	614	641	684	14.5	0.0602	0.1730	0.7045	0.8595/− 0.0649	0.2561/− 2.534

^1^THI − 21 d = temperature-humidity index calculated on the − 21 d before the calving and divided into three categories: THI < 68 as a thermal comfort zone (*n* = 31), 68–72 as mild heat stress (*n* = 35), and THI > 72 as heat stress for dairy cows (*n* = 34).

^2^AFC = age first calving.

^3^BSD = blood sampling day.

^a,b^Means of the explanatory variable within the category of THI on the − 21 d before calving within the row with different letters differed significantly (*P* ≤ 0.05).

**Table 5:** Impact of the temperature-humidity index (THI) − 14 d before calving on dairy cows’ fertility indices and immunometabolic blood indices.

Item	THI –14 d			SEM	*P-*value			*P-*value/Estimate (± 95% CI)	
	< 68	68–72	> 72		Farm (*H*_i_)	Dam parity (*L*_j_)	THI − 14 d^1^ (*B*_k_)	AFC^2^ (β_1_afc_l_)	BSD^3^ (β_2_ds_m_)
Fertility									
First estrus pp, d	50.5	69.8	77.0	1.22	0.4816	0.4395	0.6246	0.4167/0.3348	–
Calving interval, d	71.5	90.8	98.0	3.76	0.4541	0.4524	0.1968	0.1939/0.2015	–
AI period service, d	33.6	42.0	48.3	2.31	0.1957	0.2492	0.5051	0.4217/0.0417	–
Services per conception	1.60	2.00	2.30	0.15	0.1957	0.4482	0.5051	0.4217/0.0019	–
Days open, d	105	133	146	5.37	0.8012	0.9610	0.3379	0.1641/0.2433	–
Inter-calving period, d	380	408	421	6.98	0.6174	0.4427	0.4755	0.1612/0.6134	–
Immunometabolic blood indices									
Insulin, ng/mL	5.57	5.52	5.52	0.09	0.2302	0.1236	0.0552	0.6917/− 0.0009	0.3232/− 0.0149
IGF-I, ng/mL	76.3	70.6	66.2	2.27	0.0515	0.8444	0.3258	0.0130/− 0.1559	0.2844/− 0.3844
NEFA, µmol/l	56.0^b^	56.3^b^	69.4^a^	1.60	0.6329	0.3328	0.0318	0.3909/− 0.0386	0.9715/0.0097
BHB, mmol/L	5.45	7.77	8.53	0.69	0.5565	0.4853	0.4213	0.3160/− 0.0264	0.7267/0.0556
ApN, μg/mL	5.65	5.33	5.15	0.16	0.6760	0.5135	0.4249	0.0149/− 0.0141	0.4513/− 0.0249
LEP, μg/mL	1.60	1.82	1.82	0.06	0.3624	0.7571	0.1890	0.0070/− 0.0056	0.1552/− 0.0169
FSH, ng/mL	7.20	6.93	6.56	0.14	0.2406	0.3138	0.1100	0.1946/− 0.0039	0.3613/− 0.0169
LH, ng/mL	6.56	6.50	6.13	0.14	0.0549	0.2850	0.3946	0.6286/− 0.0020	0.8516/− 0.0047
TNF-α, ng/L	415^b^	467^a^	485^a^	15.6	0.0643	0.0551	0.0217	0.3874/− 0.3160	0.0568/− 4.4202
IL-1, ng/L	14.5	14.7	15.5	0.59	0.3120	0.4704	0.1892	0.0110/− 0.394	0.2325/− 0.1060
IL-6, ng/L	625	639	625	14.5	0.0628	0.0131	0.0595	0.8352/− 0.0589	0.0242/− 4.1677

^1^THI − 14 d = temperature-humidity index calculated on the − 14 d before the calving and divided into three categories: THI < 68 as a thermal comfort zone (*n* = 31), 68–72 as mild heat stress (*n* = 31), and THI > 72 as heat stress for dairy cows (*n* = 38).

^2^AFC = age first calving.

^3^BSD = blood sampling day.

^a,b^Means of the explanatory variable within the category of THI on the − 14 d before calving within the row with different letters differed significantly (*P* ≤ 0.05).

**Table 6:** Impact of the temperature-humidity index (THI) − 7 d before calving on dairy cows’ fertility indices and immunometabolic blood indices.

Item	THI –7 d			SEM	*P-value*			*P-value/Estimate (± 95% CI)*	
	< 68	68–72	> 72		Farm (*H*_i_)	Dam parity (*L*_j_)	THI − 7 d^1^ (*B*_k_)	AFC^2^ (β_1_afc_l_)	BSD^3^ (β_2_ds_m_)
Fertility									
First estrus pp, d	49.9^b^	69.3^ab^	82.0^a^	1.22	0.4118	0.6522	0.0426	0.2267/0.4823	–
Calving interval, d	70.9^b^	90.3^ab^	103^a^	3.76	0.4535	0.4477	0.0200	0.3807/0.1457	–
AI period service, d	39.5^b^	40.8^a^	59.3^a^	2.31	0.1038	0.0911	0.0378	0.4055/0.0464	–
Services per conception	1.88^b^	1.94^b^	2.82^a^	0.15	0.1038	0.0911	0.0262	0.4055/0.0022	–
Days open, d	110^b^	131^ab^	162^a^	5.37	0.7174	0.8734	0.0286	0.2964/0.1922	–
Inter-calving period, d	385^b^	406^ab^	437^a^	6.98	0.3442	0.7432	0.0184	0.2024/0.5567	–
Immunometabolic blood indices									
Insulin, ng/mL	5.48^b^	5.60^ab^	5.69^a^	0.09	0.3655	0.0509	0.0412	0.6805/− 0.0013	0.6257/− 0.0084
IGF-I, ng/mL	73.6^a^	70.1^ab^	62.1^b^	2.27	0.0289	0.6524	0.0232	0.0077/− 0.1855	0.3524/− 0.3282
NEFA, µmol/l	56.0^b^	57.9^b^	68.5^a^	1.60	0.5178	0.4352	0.0363	0.2383/0.0587	0.9599/− 0.0135
BHB, mmol/L	5.56	6.73	9.79	0.69	0.6379	0.5155	0.2164	0.3835/− 0.0261	0.9101/− 0.0184
ApN, μg/mL	5.58	5.46	4.85	0.16	0.7166	0.9019	0.2757	0.0299/− 0.0132	0.4798/− 0.0225
LEP, μg/mL	1.27	1.53	2.13	0.06	0.1895	0.5024	0.5018	0.0380/− 0.0047	0.2298/− 0.0146
FSH, ng/mL	6.38^b^	6.73^ab^	7.48^a^	0.14	0.2424	0.8589	0.0272	0.2122/− 0.0045	0.4829/− 0.0137
LH, ng/mL	6.91^a^	6.49^ab^	6.06^b^	0.14	0.0415	0.4537	0.0441	0.4128/− 0.0038	0.9213/− 0.0025
TNF-α, ng/L	421^b^	458^a^	492^a^	15.6	0.0595	0.6403	0.0332	0.8957/− 0.0691	0.1588/− 4.1991
IL-1, ng/L	12.4^b^	14.1^ab^	17.8^a^	0.59	0.0479	0.9572	0.0176	0.0177/− 0.0421	0.3774/− 0.0811
IL-6, ng/L	598^b^	636^ab^	688^a^	14.5	0.0083	0.1148	0.0192	0.7890/0.0985	0.1534/− 2.9702

^1^THI − 7 d = temperature-humidity index calculated on the − 7 d before the calving and divided into three categories: THI < 68 as a thermal comfort zone (*n* = 31), 68–72 as mild heat stress (*n* = 31), and THI > 72 as heat stress for dairy cows (*n* = 38).

^2^AFC = age first calving.

^3^BSD = blood sampling day.

^a,b^Means of the explanatory variable within the category of THI on the − 7 d before calving within the row with different letters differed significantly (*P* ≤ 0.05).

Immunometabolic blood indices were associated with evaluated parameters, including THI during the close-up dry period. An increase in THI values on the − 21, − 14, and − 7 d before the calving was linked to an increase in concentrations of NEFA and TNF-α (*P* ≤ 0.05). Additionally, an increase in THI on the − 7 d before calving was associated with higher concentrations of insulin, FSH, and biochemical blood indices related to immune response, such as IL-1 and IL-6. Conversely, it was associated with lower concentrations of IGF-I and LH (*P* ≤ 0.05). However, no significant association was observed between THI values on the − 21 and − 14 d before the calving and concentrations of all analyzed immunometabolic blood indices, except for NEFA and TNF-α.

## Discussion

Climate change has a direct and concerning impact on livestock performance and welfare. Rising temperatures and other environmental factors such as relative humidity are contributing to the occurrence of HS in livestock, making it one of the most significant problems worldwide^6^. There is substantial evidence highlighting the potential negative effects of HS on dairy herds. However, most published studies on this issue have primarily focused on its effects on lactating cows^21^, calves, and heifers^22–25^. They have also explored the effectiveness of feed additives in mitigating the negative consequences of HS^26,27^, as well as changes in farm management, including the implementation of cooling systems like a combination of ventilation and water spraying, especially during lactation^28^. Additionally, limited scientific data are available on the carry-over effects of HS occurrence defined by the THI during the close-up dry period, which is a critical time for high-yielding dairy cows during the transition period, on productive performance, fertility, and immunometabolic status during the subsequent lactation.

The current study presented prospective cohort data, and the HS occurrence model employed was similar to previous studies^15,16,29^. These studies, like ours, collected data during a single summer season, and the cows were managed under similar conditions and exposed to the same photoperiod. As a result, the observed associations in performance and other variables are likely influenced primarily by the heat challenge itself. However, it is important to note that our study was conducted as a prospective cohort study on five commercial dairy cow farms. Due to the nature of field studies, it was not possible to measure individual parameters such as DMI, body weight, and their changes. This limitation may have an impact on the results and could potentially mask certain effects. Additionally, blood samples were collected only once, at 3 weeks after calving, during routine veterinary activities. This limited sampling frequency is another constraint of our study. Nevertheless, the observed associations of physiological changes serve as a valuable observational study, providing a basis for further research in controlled conditions. This is particularly important in the context of the need to elucidate the adaptation mechanisms connecting HS and NEB occurrence with the immune response of dairy cows, especially during the still relatively less understood transition period.

Dry cows are not only susceptible to thermal stress during lactation but also during the dry period that precedes lactation. Cows exposed to HS during the dry period have been found to exhibit altered postabsorptive metabolism and produce significantly less milk during the subsequent lactation, with reductions ranging from 3 to 7.5 kg/d^8,30,31^. However, to the best of our knowledge, only limited studies have directly focused on the timing of HS occurrence during the close-up dry period and its impact on subsequent lactation performance. The results of our study suggest that the thermal status of dry cows has carry-over effects on their performance in the subsequent lactation. Specifically, dairy cows’ lactation performance was found to be associated with increasing values of THI during the close-up dry period. Cows exposed to HS (THI > 72) on − 21, − 14, and − 7 d before expected calving yielded about 2.30, 2.60, and 2.90 kg less milk, respectively. Additionally, an increase in THI on − 7 d before calving was linked to a lower concentration of IGF-I. Similarly, Menta et al.^11^ demonstrated that HS during the close-up dry period was associated with reduced milk yield in subsequent lactation, with reductions of 1.70 and 2.40 kg/d for primiparous and multiparous dairy cows, respectively. Bohmanova et al.^32^ also reported a reduction of 0.39 kg/DIM yield per unit increase in THI in temperate climates. Conversely, a study by Gomes et al.^33^ showed that active cooling applied only during the close-up phase of the dry period improved milk production by 1.40 kg/d up to 60 DIM in the subsequent lactation. However, the cellular and molecular mechanisms responsible for the observed reduction in mammary function due to HS during the close-up dry period are not yet well understood^8^. The mammary gland undergoes two phases of reconstruction during the dry period: the first phase is associated with intensive apoptosis of mammary epithelial cells, and the second phase occurs closer to calving, characterized by intensive cell proliferation and redevelopment. These processes are crucial for milk production in the subsequent lactation^34^. The activity and number of mammary epithelial cells have a direct impact on the capacity for milk synthesis, and the balance between apoptosis and cell proliferation plays a key role throughout the lactation period. HS has a negative impact on both of these processes associated with mammary gland tissue microstructure. During the close-up dry period, HS has been shown to decrease the possibility of cell proliferation and redevelopment^35^. Moreover, during the subsequent lactation, it can affect a reduced number of mammary gland follicles surrounded by larger areas of stromal connective tissue, which negatively impacts milk yield^36^. On the other hand, IGF-I, one of the most potent lactogenic hormones produced by the liver under the control of growth hormone, is considered responsible for mammary gland function and increased milk production^37^. Consistent with our study, research has reported that circulating IGF-I concentrations are moderately depressed during HS. This decrease can be attributed to reduced hepatic responsiveness to GH during HS^38^. In our study, a THI > 72, 21 d before calving, was associated with lower milk fat and protein percentages. The mechanisms responsible for the reduction in milk protein and fat content due to HS remain largely unknown, but multiple biological systems are likely involved^39^. Some studies suggest that HS has a direct effect on decreasing milk protein content rather than downregulating mammary protein synthesis or reducing feed intake^40^. Other studies suggest that heat-stressed cows may experience decreased glucose and fatty acid concentrations. This could result in increased utilization of systemic amino acids, potentially limiting the amino acid supply to the mammary gland for milk protein and fat synthesis^41^.

In our study, an increasing THI value > 72 during the close-up dry period was associated with a linear deterioration of BCS on the calving day and 21 d later, along with higher blood NEFA concentration. Furthermore, milk fat and the composition of milk fatty acids were associated with THI levels during the close-up dry period, particularly 14 and 7 d before calving. These correlations included an increase in milk fat content and concentrations of UFA and LCFA, and a decrease in levels of SCFA, SFA, and MCFA. Additionally, an increasing THI on the − 7 d before calving was associated with higher blood insulin concentration. The thermal status of cattle during the dry period has significant carry-over effects on metabolic responses in early lactation^42^. HS during the close-up dry period may cause similar changes observed during NEB occurrence in the transition period^43,44^. HS reduces body weight and BCS after calving, which may be associated with increased energy expenditure due to HS exposure and the physiological decrease in dry matter intake approaching parturition^8,30,35^. Heat-stressed dairy cows during the close-up dry period may experience NEB after calving, with increased adipose tissue mobilization, higher NEFA circulating, and enhanced insulin response at peripheral tissues due to greater insulin resistance at the systemic level after calving compared to thermoneutral dry cows^42^. This altered profile of blood metabolites suggests a decrease in BCS^45^, more active adipose tissue mobilization, greater nutrient demand for milk synthesis, and increased milk fat content in heat-stressed cows^46^. Milk fat might be synthesized from the metabolic substrates absorbed from blood or de novo in the mammary gland^47^. During early lactation, approximately 40% of NEFA serves as a source of fatty acids in milk triacylglycerols^48^. Consequently, changes in AT mobilization can significantly impact milk fat synthesis. Miller et al.^49^ found that about 56% of the variation in mammary uptake of NEFA is explained by their concentrations in arterial blood. These observations explain why long-chain fatty acids (i.e., fatty acids > 16 carbons) typically found in AT increase when NEFA levels rise in early lactation. In support of this, NEFA is positively correlated with milk fat concentration (*r* = 0.76)^50^. Mann et al.^51,52^ found that a higher blood concentration of NEFA is associated with a higher content of LCFA in the milk. This suggests that AT mobilization plays a vital role in providing substrates for milk fat synthesis, consistent with other findings^49,53^. Similarly, our results align with those of Mann et al.^51,52^ and Hammami et al.^54^, indicating that milk fat samples collected during HS tend to have higher proportions of LCFA but lower proportions of SCFA and MCFA compared to those collected under temperate conditions. However, it is worth noting that HS can also affect other factors such as pH and rumen fermentation^55^, which, in turn, can alter milk fatty acid synthesis and composition.

In the present study, an increase in the THI value, indicating the occurrence of HS, from < 68 to > 72 during the close-up dry period was associated with an increase in SCC. SCC is a widely used indicator of milk quality and indicates the likelihood of intramammary infections more than mastitis^56^. Polymorphonuclear neutrophil leukocytes, a type of white blood cell, are the primary source of the increased SCC during inflammation. The heightened risk of infection during HS can be attributed to a depressed immune function^9,56^. According to Tao et al.^57^, a compromised immune system may partly explain the increased incidence of mastitis during the summer months. Moreover, high temperatures can increase the risk of mastitis by promoting the survival and multiplication of pathogens^58^.

Dairy cattle fertility is indeed significantly influenced by a variety of environmental factors, including nutrition, herd management, and barn microclimate^59^. However, there has been limited information available concerning the fertility of cows exposed to HS during the late dry period, as compared to other phases of the transition period. In the current study, dairy cows exposed to HS (THI > 72) 7 d before calving experienced a decline in fertility as confirmed by delayed manifestation of the first estrus postpartum and an extended calving interval of approximately 32 d each, and a higher number of services per conception and an extended period of AI, days open, and inter-calving period, which increased by approximately 1.00, 20, 52, and 52 d, respectively. HS can have a negative impact on fertility, particularly affecting the reproductive tract, including the hypothalamus-pituitary-ovarian axis^60^. LH and FSH play crucial roles in regulating the formation of the corpus luteum, follicular development, and ovulation. Exposure to HS can disrupt endocrine pathways, leading to alterations in the production and secretion of ovarian hormones. This disruption can impede the final maturation and ovulation of the preovulatory follicle and affect the establishment and maintenance of pregnancy. The development of the preovulatory follicle begins months before ovulation, and both HS and diseases can have long-lasting effects on reproduction^11^. According to Torres-Júnior et al.^61^, oocytes can be damaged by HS as early as 105 d before ovulation. While there is some variation in the literature regarding the effects of HS on gonadotropins, most studies have shown that THI > 72 reduces LH secretion and the sensitivity of follicular cells to LH during maturation. This reduction can negatively impact the formation and function of the corpus luteum, and ovulation^21^, and ultimately lead to delayed manifestation of the first estrus postpartum and prolonged calving intervals^43^. On the other hand, it is important to note that HS can also lead to an increase in FSH secretion, which is associated with a higher number of follicular developments in the ovary^62^. This increase is likely due to a decrease in the inhibition concentration^60^, which may explain the significant rise in double ovulation and twin births following the summer fertilization period. Our research has revealed an association between THI > 72, 7 d before expected calving, and biochemical blood indices that influence fertility indices by decreasing LH and increasing FSH concentrations. However, the precise effects of HS on follicular growth, oocyte quality, embryo development, and survival have not been precisely defined due to the complexity of the process and the lack of accurate experimental models^63^. Early antral follicles, measuring approximately 0.5–1.0 mm in diameter, have been suggested to be sensitive to HS^64^. Dairy cows exposed to HS during the late dry period may experience reduced growth and development competence of antral follicles containing the oocyte destined to ovulate 40–60 d later. This reduction is directly associated with fertility parameters such as an increased number of services per conception and an elongated AI service period, days open, and inter-calving period^21^. Morton et al.^65^ estimated that THI > 72 for the close-up dry period and after breeding decreased the conception rate by approximately 30%, and this effect was further exacerbated by an extended period of HS, including single days near the day of breeding^66^. The negative impact of HS on reproductive performance has resulted in significant economic losses in the dairy industry^67^, primarily due to increased calving intervals and higher culling rates^68^. Therefore, based on the results of our study, implementing cooling measures for dry cows during at least the last week before the calving may help mitigate the detrimental effects of HS on the antral follicles containing the oocyte and improve fertility performance in dairy cattle herds.

In the current study, an increase in THI during the close-up dry period, specifically on − 21, − 14, and − 7 d before calving, was associated with a rise in TNF-α concentration. Additionally, an elevation in THI on the − 7 day before calving was linked to increased concentrations of biochemical blood indices, such as IL-1 and IL-6. All the above-mentioned cytokines are associated with a pro-inflammatory immune response. The impact of the stress response on the immune system remains unclear^69^, with some studies demonstrating either stimulation or suppression of the immune response depending on factors such as the type of stressor, duration of exposure to the stressor, and the animal’s condition at the time of stress^42^. HS has been shown to adversely affect the immune response by stimulating the primary and secondary lymphoid organs, leading to the production of antibodies, cytokines, and acute-phase proteins^70^. Consistent with our findings, Chen et al.^71^ reported higher concentrations of blood TNF-α, IL-1, and IL-6 in dairy cows exposed to HS compared to those in thermoneutral conditions, suggesting the possibility of an inflammatory condition resulting from the HS environment and subsequent lipolysis. Similarly, in line with our results, Trevisi et al.^72^ reported higher serum TNF-α, IL-1, and IL-6 concentrations in cows with greater body fat mobilization, as indicated by higher plasma NEFA concentrations during the transition period after calving. Mann et al.^52^ also found that the inflammatory process in adipose tissue associated with lipolysis during the transition period was characterized by the upregulated synthesis of proinflammatory cytokines, such as TNF-α, IL-1, and IL-6, which were reflected in high concentrations of NEFA.

In conclusion, lactation performance was associated with the THI during the close-up dry period. An increase in THI on the − 21, − 14, and − 7 days before calving resulted in a decrease in milk yield by approximately 2.30, 2.60, and 2.90 kg, respectively. Also, the THI on the − 7 day before calving was negatively associated with fertility. This was evident from a delayed onset of the first estrus postpartum, an elongated calving interval of approximately 32 d, an increase in the number of services per conception by 1.00, and an elongated AI service period, days open, and an inter-calving period by about 20, 52, and 52 d, respectively. Moreover, increasing THI values during the close-up dry period were associated with higher concentrations of NEFA and TNF-α in the blood. Additionally, an increase in THI values on the − 7 day before calving was linked to higher concentrations of insulin, FSH, and biochemical blood indices associated with an immune response, such as IL-1 and IL-6, as well as lower concentrations of IGF-I and LH. These findings suggest that exposing dairy cows to HS during the close-up dry period has a negative impact on their productive performance, fertility, and immunometabolic blood indices in the subsequent lactation. Therefore, implementing management changes on the farm to mitigate the negative effects of HS during the close-up dry period is crucial. However, it is important to note that the above-observed associations are based on our pilot study, highlighting the need for further precise research in controlled condition trials.

## Methods

### Farms

The study was carried out from July to September 2018 on five commercial dairy farms that housed Polish Holstein–Friesian cows in northwest Poland (Table 7). Prospective cohort data were gathered from these farms. The selection criteria for these herds included a 305-d milk yield of over 9500 kg, a farm size of more than 80 dairy cows, housing exclusively in free-stall barns, utilization of a total mixed ration (TMR) feeding system, and a dry period (56 d).

**Table 7:** General farms and dairy cows’ characteristics.

Item	Farms				
	1	2	3	4	5
General farms information					
No. cows in herds	85	110	94	88	86
No. primiparous	30	33	34	26	26
Average AFC^1^	734	754	790	774	789
Average parity no	3.4	2.8	2.8	2.6	2.8
Average 305-d milk yield (kg)	10.021	10.640	9.987	10.342	9.875
General tested cows’ information					
No. tested cows	20	20	20	20	20
No. primiparous	8	8	9	8	8
Average AFC	736	777	774	752	769
Average parity no	2.9	2.8	2.4	2.8	2.6
Average 305-d milk yield (kg)	10.800	10.455	9.252	10.116	9.650
Dry period length (d)	56	56	57	56	56
Predicted close-up dry period length (d)	21	21	21	21	21
Number of calving per month					
July	6	5	9	3	5
August	11	8	6	5	8
September	3	7	5	12	7

^1^AFC—the age at first calving.

### Animals

The part of the animal trial of heat stress has been reported also previously ^16^. The dataset utilized in this study consisted of 100 clinically healthy Polish Holstein–Friesian dairy cows, selected based on parity (2.4–2.9; average 2.7) and 305-d milk yield (9,252–10,800 kg; average 10,055 kg). Twenty animals were chosen from each of the five farms participating in the study. Before dry-off, all cows were managed according to similar standard dairy farm protocols. The cows were dried off 56 d (± 1) before their expected calving date, and data recording for the prospective cohort commenced during the close-up dry period (beginning 3 weeks before calving) and continued until 150 d in milk (DIM). The calving of the first and last cows in the study occurred 42 calendar days apart. The herds maintained similar management and housing practices. During the dry period, the cows were housed in shaded free-stall barns with natural ventilation and bedding consisting of chopped straw. After calving, all cows received similar treatment, including access to cooling measures such as shade and fans or air mixers during the lactation phase. The fans or air mixers were automatically activated when the ambient temperature exceeded 21.0 °C, and they maintained an air velocity of 1.8 m/s, following the guidelines of Bailey et al. ^73^. The ambient photoperiod was consistent across all farms, with metal halide lighting installed over the stall areas. These lights were manually controlled to provide approximately 230 lx and a lighting duration of approximately 14 h of light and 10 h of dark. Specifically, the lights were turned on from 2000 to 0600 h. During the close-up dry period, cows were housed in group pens within the free-stall barn building, accommodating up to 10 cows/pen, with dimensions of 4 m × 12 m × 1.5 m (length × width × height). When cows displayed signs of parturition, they were transferred to individual maternity pens with straw bedding within the same building. There was a separate group for early lactation cows from 2 to 21 DIM, with an average group size of 10 cows and a stocking density of < 1 cow/lair. Lactating cows were housed in pens designed for 20 to 40 cows, featuring individual open-ended cubicles with chopped straw bedding and concrete slatted floors in the alleyways.

Representative samples of forages, including corn, grass, and alfalfa silages, were collected monthly. These samples were analyzed using the near-infrared reflectance spectroscopy method as per PN-EN 12,099:2017-10, employing a FOSS DS3™ (Foss Electric, Hillerod, Denmark). The analysis covered various nutritional components, including dry matter, crude protein, neutral detergent fiber, acid detergent fiber, ether extract, and starch. This analysis was conducted consistently throughout the experiment. The nutritional value of the feed components was calculated using the nutrient content obtained through analysis. These calculations were performed using the AMTS.Cattle.Pro version 4.7 (2017, AMTS LLC, Groton, NY). The formulated diets for both close-up dry and lactating cows were adjusted monthly to meet the nutritional requirements following the guidelines set forth by the National Research Council^74^. These diets were composed primarily of silages such as corn, grass, and alfalfa, as well as concentrates such as soybean meal, rapeseed meal, barley, and triticale. A mineral–vitamin premix was also included. These diets were delivered to the cows twice daily, at 0600 and 1400 h, and were made available ad libitum. Close-up dry cows were provided with a TMR diet from 21 d prepartum until calving, with an average feed intake of 12 kg dry matter intake (DMI). After calving, all cows were transitioned to an early FRESH lactation diet up to 21 DIM, with an average feed intake of 24 kg dry matter intake (DMI), and 35 kg milk yield. Subsequently, from 22 to 150 DIM, they were fed a TMR I lactation diet, formulated to meet the nutritional requirements of dairy cows consuming 28 kg DMI and producing 40 kg of milk. The close-up diet contained a range of 65.1–81,9% forage, 14.6–15.5% CP, 35.1–39.0% NDF, and 15.9–18.5% starch, and (− 45.0)–(− 68.0) mEq/kg dietary cation–anion balance (DCAB) on a DM basis. The early lactation FRESH diet contained a range of 50.8–65.2% forage, 17.5–18.1% CP, 31.1–34.1% NDF, 20.7–24.4% starch, and 255–286 mEq/kg DCAB on a DM basis. The TMR I diet contained a range of 45.5–54.9% forage, 15.9–17.5% CP, 28.1–31.5% NDF, 25.5–28.4% starch, and 268–320 mEq/kg DCAB on a DM basis. The composition and nutritional value of close-up and lactation diets are summarized in Supplemental Table S1. Orts, which refer to leftover feed, were removed daily before the morning feeding. Throughout the experiment, water was supplied to the cows ad libitum and was accessible through self-filling troughs. The body condition score of the cows, rated on a scale of 1 (thin) to 5 (fat) according to Edmonson et al.^75^ was recorded on the calving day and again 3 weeks after calving (on d 21 ± 3).

### Animal health and numbers

Throughout the entire experimental period, a veterinarian conducted daily health checks on the cows to ensure their well-being. The cows received three vaccinations for rotavirus and coronavirus, administered approximately 60 and 30 days before calving, with an additional vaccination given 2 weeks after calving. For early fresh lactation cows, routine daily monitoring practices were in place. This included assessing body temperature on d 1 and d 5 after calving. Additionally, health status was monitored for various conditions, including hypocalcemia, ketosis, dystocia, retained placenta, claw health, and mastitis. Out of the initial group of 100 Polish Holstein–Friesian dairy cows, six were excluded from the study after calving, within the first 60 d post calving. This exclusion was due to health complications/disease (*n* = 4) and mastitis (*n* = 2). Data collected from these animals were included in the analysis up to the point of their removal from the study.

### Environmental data

The AT and RH within the barn were continually monitored at 15-min intervals using data loggers (WatchDog A-Series, SpecWare 9 Basic, Spectrim Technologies Inc., Aurora, USA). These data loggers had a temperature range of − 45 to 85 °C with an accuracy of ± 0.6 °C, and a humidity range of 0% to 100% with an accuracy of ± 3%. They were strategically positioned inside the building, approximately 3 m above the area where the cows were housed. This positioning aligns with the recommendations put forth by Hut et al.^15^ and Lambertz et al.^29^. To assess the THI, the following formula was used, as recommended by the National Research Council^76^: THI = (1.8 × *T* + 32) − [(0.55 − 0.0055 × RH) × (1.8 × *T* − 26)]. Here, T represents the air temperature (°C), and RH stands for RH (%). In this particular study, the cows were categorized based on their THI exposure during the close-up dry period. The daily THI values at 21, 14, and 7 days before the calving were calculated and these values were divided into three distinct categories: THI < 68 as a thermal comfort zone, 68–72 as mild HS, and THI > 72 as HS for dairy cows. These specific THI categories and the accompanying equation were selected because they have been used in prior dairy cow trials conducted in a temperate climate^9,16^.

### Productive performance

Dairy cows were milked twice daily at 0500 and 1700 h, starting from the day of calving until 150 DIM. Their milk yield (kg) was electronically recorded. Milk samples (15 mL) were collected monthly from both morning and afternoon milkings and placed into specialized tubes with potassium dichromate as a preservative. The milk samples underwent evaluation by the Polish Federation of Cattle Breeding and Dairy Farmers. The analysis of the milk samples included assessments of protein content (%), milk urea (MU, mg/mL), fat content (%), and milk fatty acids (FAs, g/100 g of fat) using the Fourier transform infrared (FTIR) spectroscopy method according to PN-ISO 9622:2015–09. This analysis was conducted using equipment from Foss Electric (Hillerod, Denmark), specifically the MilkoScan FT 6000, along with diamond cuvettes (Foss, Hillerod, Denmark). The somatic cell count (SCC, 10^3^ cells/mL) was determined using a Foss-o-Matic FC instrument (FOSS Electric, Hillerød, Denmark) following PN-EN 13366-2:2008. The FTIR absorption spectra encompassed 1,060 infrared frequencies (wavenumbers) spanning from 925 to 5,008/cm, representing the milk samples’ infrared light absorption characteristics. The FAs traits, expressed as g/100 g of fat, comprised four individual FAs, namely C14:0, C16:0, C18:0, and C18:1, as well as eight groups, namely saturated FAs (SFAs), unsaturated FAs (UFAs), monounsaturated FAs (MUFAs), polyunsaturated FAs (PUFAs), trans unsaturated FAs (TRANSFAs), short-chain FAs (SCFAs), medium-chain FAs (MCFAs), and long-chain FAs (LCFAs). Additionally, three ratios were calculated: the MCFA to SCFA ratio (MCFA/SCFA), the SFA to UFA ratio (SFA/UFA), and the C18 desaturation index (DI18). DI18 was determined by following the formula proposed by Komisarek et al.^55^, which involves calculating the proportion of C18:1 to the sum of C18:0 and C18:1, and then multiplying the result by 100.

### Fertility performance

Starting approximately 28 d (± 3) after calving, a veterinarian conducted weekly transrectal ultrasonography to assess the ovarian structures of the cows. This evaluation was performed using a color Doppler ultrasound scanner (SSD-5500, Aloka Co., Japan) equipped with a 7.5 MHz convex transducer (UST-995-7.5, Aloka Co., Japan). The veterinarian scanned each ovary from multiple angles to thoroughly assess the position of the structures. The confirmation of the first estrus post-calving was based on the presence of a visible corpus luteum (dominant follicle measuring ≥ 10 mm in diameter) in either ovary. Subsequently, the cows were examined weekly until the second estrus was confirmed. Day 1 of the estrus cycle was designated as the day when the new estrus was confirmed. This day marked the period between the disappearance of a dominant follicle measuring ≥ 10 mm in diameter and the appearance of a new corpus luteum in the same position. Cows exhibiting signs of estrus were artificially inseminated (AI). Pregnancy was diagnosed by transrectal ultrasonography on d 30 (± 3) after AI. The fertility of dairy cows was evaluated based on several indices such as the first estrus postpartum (number of days from calving to the first estrus), calving interval (number of days from calving to the first service), insemination period service (days from the first AI service to conception), services per conception (number of AI services required for conception), days open (number of days from calving to the next fertilization), and inter-calving period (number of days from one calving to the next calving).

### Blood sampling and analyses

Blood samples were collected from each cow approximately 4 ± 0.5 h after morning feeding, precisely 3 weeks after calving (on d 21 ± 3) during standard veterinary procedures. The samples were obtained from the tail vein using a 10 mL vacutainer designed for serum collection (KABE, Poznan, Poland). Following collection, the vacutainers containing the blood samples were subjected to centrifugation at 3000 × *g* for 15 min at 4 °C. This process allowed for the isolation of serum, which was then stored at − 20 °C until further analysis. The serum samples were utilized to determine the concentrations of insulin, insulin-like growth factor-I (IGF-I), NEFA, adiponectin (ApN), leptin (LEP), follicle-stimulating hormone (FSH), luteinizing hormone (LH), TNF-α, IL-1, and IL-6 using bovine ELISA kits (Shanghai Sunredbio Technology, China). All assays were performed in duplicate, and the absorbance values were measured at 450 nm using a microplate spectrophotometer (Synergy 2, BioTek, BIOKOM, Warsaw, Poland). Specifications of bovine ELISA kits from Shanghai Sunredbio Technology (China) used for determining hormones, cytokines, and metabolic blood indices were reported previously^16^. Additionally, the concentration of β-hydroxybutyrate (BHB) was measured using a bovine kit manufactured by Pointe Scientific (Warsaw, Poland). The BHB assay range was 0.05 to 6.9 mmol/L (catalog no. H7587), and absorbance values were read at 340 nm using the colorimetric method and the same microplate spectrophotometer (Synergy 2, BioTek, BIOKOM, Warsaw, Poland). Quality control measures were implemented to ensure the reliability of the results, with both interassay and intraassay variation kept within a coefficient of variation of ≤ 5% for all blood variables.

### Statistical analyses

Before analysis the PROC MEANS was used and then all data underwent normality screening using PROC UNIVARIATE in SAS version 9.4, SAS Institute^77^. Fertility parameters, such as service per conception and AI period service were subjected to box-cox transformation, and milk SCC a logistic transformation function before statistical analysis. The results for the above fertility parameters and milk SCC were presented in Tables as means before a transformation, but the significance between the explanatory variables for these indices after transformation. The explanatory variables for THI during the close-up dry period (− 21, − 14, and − 7 d) were divided into three categories, which were consistent for the analysis of productive performance, fertility, and immunometabolic blood indices. The effect of HS, defined by the THI during the close-up dry period on productive performance, fertility, and immunometabolic blood indices was determined using the GLM procedure of SAS separately for each of the explanatory variables (THI on the − 21, − 14, and − 7 d before calving) under the models described in the equations. Productive performance was analyzed using the following model: *Y*_ijklmn_ = μ + *H*_i_ + *L*_j_ + *B*_k_ + β_1_afc_l_ + β_2_dl_m_ + *e*_ijklmn_, where *Y*_ijklmn_ is the value of the dependent variable, μ is the overall mean, *H*_*i*_ is the fixed effect of the farm (*i* = 1, 2, 3, 4, 5), *L*_*j*_ is the fixed effect of parity of dam (*j* = 1, 2), *B*_k_ is the explanatory variable—fixed effect of the category of THI on the − 21, or − 14, or − 7 d during the close-up dry period (*k* = 1, 2, 3; listed in Table 8), β_1_ and β_2_ are the partial linear regression coefficients, afc_l_ is age at first calving, dl_m_ is DIM, and *e*_ijklmn_ is the random error. The fertility indices were analyzed using the same statistical model but without the second partial regression coefficient (β_2_dl_m_). The immunometabolic blood indices were analyzed using the following model: *Y*_ijklmn_ = μ + *H*_i_ + *L*_j_ + *B*_k_ + β_1_afc_l_ + β_2_ds_m_ + *e*_ijklmn_, where *Y*_ijklmn_ is the value of the dependent variable, μ is the overall mean, *H*_i_ is the fixed effect of the farm (*i* = 1, 2, 3, 4, 5), *L*_*j*_ is the fixed effect of parity of dam (*j* = 1, 2), *B*_k_ is the explanatory variable—fixed effect of the category of THI on the − 21, or − 14, or − 7 d during the close-up dry period (*k* = 1, 2, 3), β_1_ and β_2_ are the partial linear regression coefficients, afc_l_ is the age at first calving, ds_m_ is the day of blood sampling, and *e*_ijklmn_ is the random error.

**Table 8:** Descriptive statistics of evaluated parameters (explanatory variables) during the close-up dry period of dairy cows.

Item^1^	n	Mean	Minimum	Maximum	SEM
THI − 21 d	100	64.1	51.6	77.2	2.68
THI − 14 d	100	66.6	52.5	79.4	2.53
THI − 7 d	100	67.3	52.9	78.1	2.40

^1^THI − 21 d = temperature-humidity index calculated on the − 21 d before the calving and divided into three categories: THI < 68 as a thermal comfort zone (*n* = 31), 68–72 as mild heat stress (*n* = 35), and THI > 72 as heat stress for dairy cows (*n* = 34); THI − 14 d = temperature-humidity index calculated on the − 14 d before the calving and divided into three categories: THI < 68 as a thermal comfort zone (*n* = 31), 68–72 as mild heat stress (*n* = 31), and THI > 72 as heat stress for dairy cows (*n* = 38); THI − 7 d = temperature-humidity index calculated on the − 7 d before the calving and divided into three categories: THI < 68 as a thermal comfort zone (*n* = 31), 68–72 as mild heat stress (*n* = 31), and THI > 72 as heat stress for dairy cows (*n* = 38).

When the explanatory variables (categories of THI on − 21, or − 14, or –7 d during the close-up dry period) showed significance, individual comparisons were conducted using Duncan’s adjustment. Statistical significance was declared when *P* ≤ 0.05, and trends were noted when 0.05 < *P* ≤ 0.1.

## Supplementary Information

Supplementary Table S1.

## Data Availability

The experimental datasets of the present study can be obtained from the corresponding author on reasonable request.
