# Supplementary material for: Impact of heat stress during close-up dry period on performance, fertility and immunometabolic blood indices of dairy cows: prospective cohort study
Source: Sci Rep. 2024 Sep 11;14:21211. doi: 10.1038/s41598-024-72294-2 (PMC11390746; doi:10.1038/s41598-024-72294-2)
Supplement: Supplementary file 1 — Supplementary Table S1. [file 41598_2024_72294_MOESM1_ESM.doc]

**Supplemental Table S1**. Ingredient and nutritional value of close-up and lactation diets on a DM basis.

| Item | Farm | | | | | | | | | | | | | | |
| --- | --- | --- | --- | --- | --- | --- | --- | --- | --- | --- | --- | --- | --- | --- | --- |
|  | 1 | | | 2 | | | 3 | | | 4 | | | 5 | | |
|  | Close-up | Fresh | TMR I | Close-up | Fresh | TMR I | Close-up | Fresh | TMR I | Close-up | Fresh | TMR I | Close-up | Fresh | TMR I |
| Ingredient (%) | | | | | | | | | |  |  |  |  |  |  |
| Corn silage | 28.7 | 35.2 | 38.0 | 32.7 | 30.5 | 33.0 | 29.6 | 29.5 | 28.3 | 27.5 | 36.1 | 38.0 | 28.6 | 34.6 | 36.2 |
| Grass silage | 21.0 | 10.5 | 8.60 | 27.6 | 9.50 |  | 20.3 | 8.20 |  | 25.4 | 7.50 | 2.20 | 17.5 | 7.20 | 5.50 |
| Alfalfa silage |  | 7.45 | 5.20 |  | 15.9 | 8.40 |  | 13.0 | 13.4 |  | 11.8 | 9.00 |  | 7.50 | 9.00 |
| Hay | 15.4 | 1.00 |  | 21.6 |  | 2.40 | 22.4 |  |  | 19.4 |  |  | 18.5 |  |  |
| Straw |  |  |  |  | 9.30 | 1.70 |  | 8.20 | 6.20 |  | 5.80 | 2.60 | 3.50 | 1.50 | 4.20 |
| Corn grain silage | 5.10 | 4.80 | 5.50 |  | 4.50 | 8.10 | 5.10 | 5.80 | 9.70 | 5.10 | 5.00 | 7.50 | 5.10 | 5.20 | 5.50 |
| Sugar beet pulp |  | 8.90 | 11.5 | 13.9 |  | 5.50 |  | 8.10 | 5.10 |  | 7.00 | 7.40 |  | 8.00 |  |
| Soybean meal | 2.20 | 5.00 | 3.50 |  | 7.90 | 9.10 | 2.50 | 6.10 | 6.10 | 2.60 | 6.00 | 5.60 | 1.80 | 6.20 | 5.60 |
| Rapeseed meal | 7.50 | 5.50 | 6.20 |  | 4.20 | 9.20 | 7.50 | 3.30 | 6.40 | 7.50 | 4.50 | 7.20 | 7.50 | 4.60 | 7.30 |
| Barley grain | 1.10 | 1.10 | 2.00 |  | 5.30 | 5.10 | 2.00 | 4.10 | 9.70 | 1.50 | 3.50 | 5.30 |  | 3.00 | 5.70 |
| Triticale grain | 5.00 | 3.25 | 3.40 |  |  | 5.00 | 5.00 |  |  | 5.00 | 2.50 | 2.90 | 5.00 | 2.30 | 3.50 |
| Corn grain |  |  |  |  | 4.70 | 6.80 |  | 5.90 | 6.90 |  | 3.50 | 4.60 |  | 4.70 | 5.60 |
| Glycerol | 1.50 | 1.20 |  |  | 4.50 |  | 1.50 | 3.40 | 2.60 | 1.50 | 1.50 | 1.80 | 1.50 | 1.60 | 1.50 |
| Inert fat |  |  | 2.00 |  |  | 1.90 |  |  | 1.10 |  |  | 1.50 |  |  | 1.50 |
| Water | 8.50 | 8.30 | 8.10 |  |  |  |  |  |  |  |  |  | 6.50 | 8.30 | 4.50 |
| Minerals and vitamins^2^ | 4.00 | 7.80 | 5.00 | 4.20 | 3.70 | 3.80 | 4.10 | 4.40 | 4.50 | 4.50 | 5.30 | 4.40 | 4.50 | 5.30 | 4.40 |
| Nutrients composition^3^ (%) | | |  |  |  |  |  |  |  |  |  |  |  |  |  |
| DM as feed | 46.0 ± 0.46 | 44.3±0.54 | 44.9±0.56 | 49.2±0.44 | 49.3±0.36 | 45.2±0.34 | 45.0 ± 0.46 | 44.2±0.32 | 42.0±0.38 | 42.0 ± 0.46 | 41.3±0.54 | 43.9±0.56 | 44.0 ± 0.46 | 46.3±0.54 | 44.9±0.56 |
| CP (%) | 14.6 ± 0.12 | 17.5 ± 0.18 | 16.5 ± 0.14 | 14.9±0.14 | 17.8±0.12 | 17.1±0.16 | 15.0 ± 0.12 | 17.6±0.24 | 15.9±0.18 | 15.1 ± 0.12 | 18.1 ± 0.18 | 17.5 ± 0.14 | 15.5 ± 0.12 | 17.9 ± 0.18 | 16.5 ± 0.14 |
| NDF (%) | 37.1 ± 0.26 | 33.1 ± 0.28 | 31.5 ± 0.18 | 39.0±0.12 | 31.3±0.16 | 28.1±0.22 | 38.1 ± 0.26 | 32.3±0.28 | 30.1±0.14 | 36.1 ± 0.26 | 34.1 ± 0.28 | 30.5 ± 0.18 | 35.1 ± 0.26 | 33.1 ± 0.28 | 29.5 ± 0.18 |
| ADF (%) | 29.3 ± 0.22 | 19.6 ± 0.14 | 20.8 ± 0.14 | 23.7±0.18 | 20.3±0.12 | 17.3±0.14 | 29.3 ± 0.22 | 20.2±0.12 | 17.8±0.23 | 29.3 ± 0.22 | 19.6 ± 0.14 | 20.8 ± 0.14 | 29.3 ± 0.22 | 19.6 ± 0.14 | 20.8 ± 0.14 |
| Starch (%) | 18.5 ± 0.24 | 24.4 ± 0.32 | 28.4 ± 0.24 | 16.6±0.12 | 20.7±0.21 | 25.7±0.14 | 15.9 ± 0.24 | 20.7±0.24 | 25.5±0.24 | 17.5 ± 0.32 | 24.4 ± 0.32 | 28.4 ± 0.24 | 18.1 ± 0.24 | 24.4 ± 0.32 | 28.4 ± 0.24 |
| Ether extract (%) | 2.70 ± 0.11 | 3.60 ± 0.08 | 4.30 ± 0.10 | 2.50±0.08 | 2.20±0.06 | 2.90±0.10 | 2.70 ± 0.11 | 2.20±0.14 | 3.30±0.10 | 2.70 ± 0.11 | 3.60 ± 0.08 | 4.30 ± 0.10 | 2.70 ± 0.11 | 3.60 ± 0.08 | 4.30 ± 0.10 |
| Ca (%) | 0.60±0.01 | 0.73±0.02 | 1.06±0.01 | 0.64±0.01 | 0.75±0.04 | 0.95±0.04 | 0.58±0.02 | 0.83±0.04 | 0.94±0.08 | 0.71±0.02 | 0.82±0.02 | 0.84±0.01 | 0.54±0.01 | 0.74±0.01 | 0.92±0.04 |
| P (%) | 0.29±0.01 | 0.41±0.01 | 0.46±0.02 | 0.29±0.01 | 0.42±0.01 | 0.43±0.01 | 0.31±0.02 | 0.40±0.01 | 0.46±0.02 | 0.29±0.01 | 0.38±0.02 | 0.42±0.02 | 0.30±0.01 | 0.43±0.01 | 0.49±0.01 |
| K (%) | 1.22±0.09 | 1.37±0.11 | 1.47±0.07 | 1.28±0.14 | 1.60±0.08 | 1.48±0.08 | 1.04±0.06 | 1.34±0.08 | 1.64±0.06 | 1.08±0.09 | 1.34±0.11 | 1.55±0.08 | 1.14±0.04 | 1.46±0.07 | 1.48±0.07 |
| DCAB^4^  (mEq/kg DM) | -45±4.24 | 265±8.22 | 268±6.14 | -55±2.32 | 280±4.57 | 305±6.48 | -48±3.34 | 286±6.43 | 315±6.24 | -65±5.42 | 255±6.51 | 294±7.46 | -68±3.54 | 274±4.36 | 320±8.32 |

^1^Diets – Close-up (the feeding period from 3 wk. before calving to calving day, during the close-up period 250 g anionic salt supplementation); Fresh (the feeding period from calving to 21 DIM; 35 kg milk); TMR I (the feeding period from 22 to 150 DIM; 40 kg milk)

^2^Minerals and vitamins – 21.5% Ca, 4.0% P, 6.5% Na, 5.5% Mg, 1.200 mg/kg Cu, 4.000 mg/kg Mn, 15 mg/kg Co, 10.000 mg/kg Zn, 60 mg/kg Se, 1.200.000 jm/kg vitamin A, 180.000 jm/kg vitamin D, 6.000 jm/kg vitamin E.

^3^Nutrient concentration – representative samples of foragers such as corn, grass, and alfalfa silages were sampled monthly and analyzed using near-infrared reflectance spectroscopy (NIRS) to determine their nutritional value. The diets were formulated monthly according to the NRC (2001) requirements.

^4^Calculated as follows: DCAB (mEq/kg DM) = [(mEq of K) + (mEq of Na)] –[(mEq of Cl) (mEq of S)
